# Personality metatraits predict resilience among family caregivers responsible for a dependent youth’s chronic respiratory management

**DOI:** 10.1186/s40359-022-00791-y

**Published:** 2022-04-01

**Authors:** Sidai Dong, Timothy R. Elliott, Wen Luo, Ann Marie Warren, Robert Warren

**Affiliations:** 1 Department of Educational Psychology, Texas A&M University (4225 TAMU), College Station, TX 77843-4225 USA; 2 Baylor Scott and White Research Institute, Baylor University Medical Center, Dallas, TX USA; 3 University of Arkansas for Medical Sciences, Little Rock, AR USA

**Keywords:** Family caregiver, Chronic respiratory management, Resilience, Quality of life, Personality

## Abstract

**Background:**

Family caregivers of children and youth with severe neurodisabilities that require chronic respiratory management often report a compromised quality of life. In this cross-sectional study, we used DeYoung’s (Psychol Inq 21(1): 26–33, 2010. 10.1080/10478401003648674) conceptualization of two personality metatraits, Alpha and Beta, to test their theorized role in facilitating resilience among these family caregivers. We expected higher Alpha and Beta would exhibit direct, beneficial effects on caregiver mental and physical health quality of life (QoL), and they would operate through self-reported resilience and coping to exert positive, indirect effects on caregiver QoL.

**Methods:**

Family caregivers of children and youth at an outpatient chronic respiratory management clinic were informed of the study. Of the 68 who consented, 61 provided complete data on measures of personality traits, coping styles, and physical and mental health-related QoL. Factor analytic techniques verified the two personality metatraits, consistent with the DeYoung model. The metatraits were then used as predictor variables in a path model to predict physical and mental health-related QoL. Self-reported resilience and a coping variable were examined as possible mediators of the personality-QoL relationship.

**Results:**

Correlational analyses isolated a coping variable that met criteria as a possible mediator. The path model exhibited good fit to the data. The Alpha metatrait—characterized by emotional stability, self-regulation, perseverance, and intrinsic motivation—was directly predictive of caregiver mental health. The Beta metatrait, reflecting a disposition for adaptive flexibility, responsiveness, and interpersonal initiative, demonstrated significant indirect effects to physical and mental health through its positive association with coping efforts to maintain social support and a sense of self.

**Conclusions:**

Consistent with DeYoung’s conceptualization, higher Alpha and Beta predicted caregiver resilience, albeit through different pathways. The emotional stability, perseverance and emotional regulation associated with Alpha likely accounted for its positive association with caregiver mental health. Beta, in contrast, may operate through their adaptive flexibility, personal resourcefulness and social engagement to augment coping efforts that involve others and support family activities, which, in turn, promote their own physical and mental health. Limitations of the cross-sectional design, and potential theoretical and clinical implications of the personality metatraits and their relation to resilience are discussed.

**Supplementary Information:**

The online version contains supplementary material available at 10.1186/s40359-022-00791-y.

## Background

Children and youth with severe neurodisabilities often experience a wide range of secondary medical and physical complications and a loss of vital organ functions, often necessitating a dependence on medical technology that must be monitored and maintained by a parent or designated caregiver [2]. They are the second most ordinary pediatric users of home oxygen, many requiring ongoing assistive ventilator support, and respiratory complications are the leading cause of premature death [3, 4]. These individuals may require 24-h support and supervision because they cannot breathe without assistance, communicate, have meaningful social interaction, or ambulate. Consequently, home care is the best option for these families [5, 6].

In-home assistance for technology-dependent children and youth requires a strict adherence to therapeutic regimens, and maintenance of hygiene and cleanliness [7, 8]. These responsibilities are typically assumed by mothers [9], who are at risk for social isolation, financial adversity, disruptions in family routines, and caregiver burden should they lack appropriate formal and informal supports [10]. Many experience problems with depression, anxiety and a compromised quality of life [11–14]. Clinical guidelines from the Canadian Thoracic Society for pediatric home mechanical ventilation concluded that greater attention to the health and well-being of these caregivers is warranted, and recommended that the “…characteristics and circumstances that enable resiliency” be identified among these family caregivers so programs can help them capitalize on their strengths and cope effectively [8].

The lived experience of these family caregivers challenges prevailing notions of resilience that define the concept as an ability to “bounce back” from a major life event [15]. Although there is no real consensus in defining the concept and the mechanisms through which it facilitates adjustment, most definitions (and corresponding measures) construe resilience in the context of a stressful or “potentially traumatic” event or circumstance [16]. Unlike these conditions, family caregiving involves a daily commitment in which a person manages everyday tasks and family routines while simultaneously observing therapeutic regimens for the care recipient, and being alert to and managing sudden or stress-provoking problems that may occur. Nevertheless, some families successfully incorporate these activities into everyday routines, develop interdependent ways to share responsibilities, navigate and secure available supports, and, in some cases, eschew labels of “caregiver” and “care recipient” in favor of traditional family designations (e.g., mother, child; [17]). These family caregivers likely possess characteristics and display a level of adjustment that personify resilience.

Relevant research suggests that parents who report less distress and higher quality of life (QoL) in these chronic care scenarios are distinguished by their use of social support and specific coping skills that serve to maintain family stability and functions [12–14, 18]. Non-pathological personality traits appear instrumental in facilitating caregiver QoL, including predictable associations between neuroticism and distress (e.g., depression, anxiety; [19]). Extraversion and agreeableness are positively associated with indicators of caregiver QoL (e.g., subjective well-being and life satisfaction, respectively; [20, 21]). Evidence from the larger extant caregiver literature indicates all of the “Five Factor” personality traits—neuroticism, agreeableness, openness to experience, extraversion, and conscientiousness [22]—may correlate in expected directions with caregiver health-related QoL, and these traits are similarly associated with caregiver self-efficacy [23].

From our perspective, these personality traits are fundamental to our understanding of caregiver resilience under the routine and stressful conditions that typify chronic care scenarios. There are theoretical reasons why and how these traits pertain to individual resilience. In one of the earliest conceptualizations of resilience and personality, Block and Block’s [24] developmental model of ego control and ego resilience integrated the recognized dynamics of healthy attachments with Lewin’s [25] concepts of Permeability and Elasticity. In their conceptualization, these constructs are instrumental in effective, resourceful adaptation to transition, change, conflict and growth. Thoughtful scholars studying the way the Block constructs were described and assessed concluded that a resilient personality prototype could be characterized by a distinct pattern in five personality traits: low Neuroticism (N), and above-average scores on Extraversion (E), Openness to Experience (O), Agreeableness (A), and Conscientiousness (C). Systematic studies of this configuration found a resilient personality prototype is associated with prosocial and self-regulatory behavior, greater cognitive flexibility, increased engagement in goal-directed activities, and more optimal physical and mental health than those who do not have this personality prototype [26–28].

DeYoung [1] recognized the parallels between the Block conceptualization of a resilient personality prototype and the two overarching meta-traits, Alpha and Beta, that emerged from Digman’s [22] factor analytic studies of the five personality traits. Alpha is characterized by higher Conscientiousness and Agreeableness, and lower Neuroticism; Beta is characterized by higher Extraversion and Openness to Experience. Similar to the Block model, these two metatraits appear to embody Lewin’s [25] concepts of permeability and elasticity. Alpha (i.e., permeability) is the component through which an individual regulates basic needs to maintain stability in emotional, motivational, and social functioning, and it accomplishes this, in part, by imbuing an individual with perseverance, intrinsic motivations, and self-regulatory abilities to delay gratification to facilitate emotional stability [29]. Beta (i.e., elasticity) is the capacity to flexibly adapt to environmental demands and pressures, and a willingness to engage in new experiences and explore the environment (including relationships), reflecting a sense of initiative in personal relationships, and learn and integrate new information to maintain a sense of self. Accumulating evidence finds the metatraits are predictably associated with an array of behaviors and outcomes; however, much of this work has focused on relationship with indicators of distress [30]. Recent research finds they predict well-being in expected directions [31].

To the best of our knowledge, the two metatraits have not been included in contemporary studies of resilience. The positive qualities associated with higher Alpha and Beta metatraits should facilitate resilience among family caregivers. We conducted the present study to (a) identify the two metatraits in a sample of family caregivers of children and youth receiving chronic respiratory management, and (b) test our expectation that these would predict two QoL outcomes among these caregivers. Based on our understanding of these metatraits, we expected both would be positively associated with caregiver physical and mental health QoL, and that they would also operate through effective coping and positive appraisals of their own resilience to positively influence caregiver QoL. In this manner, our model could reveal some of the mechanisms through which these metatraits facilitate caregiver adjustment, providing us with important information about how caregivers may be resilient that, in turn, may help guide services to assist those who are not.

## Methodology

### Participants

68 family caregivers of children seen at a respiratory outpatient clinic participated in the study. Children seen at the clinic have chronic pulmonary symptoms that require daily respiratory care plans and each child must have a need for a respiratory therapy device (e.g., a ThAIRpy Vest, Emerson In-Exsufflator). Children and youth served by the clinic present with over 60 different congenital neurological diagnoses, and patients with acquired neurodisabilities such as those resulting from birth trauma and traumatic-onset disability (e.g., shaken baby syndrome; traumatic brain injury). The age range of the clinic patient base is 3 months to 32 years of age. The respiratory care needs of all clinic patients are comprehensive and complex, requiring daily respiratory care plans. Among ventilator-dependent children, 24-h care may be required due to a combination of symptoms, medication management, continuous bolus feeds, control of daily seizures, and breathing treatments which may occur up to four times a day, with individual treatment times extending from 45 min to 1 h. Individualized respiratory care in the clinic intends to achieve a degree of stability to prevent acute medical crises, and reduce or eliminate the occurrence of emergency room visits and hospitalizations.

The research project was approved by the Institution Review Boards at the University of Arkansas for Medical Sciences and Texas A&M University. Potential caregiver participants for the study were identified from the clinic database. Some caregivers were contacted prior to the interview either by telephone or letter. Others learned about the study at the time of their child’s regularly scheduled clinic visit. Participants were self-identified as a primary caregiver of a patient at the clinic. To be eligible caregivers had to be at least 18 years of age, and be able to read and write in English.

Participants received a packet that contained all research questionnaires and an information form to obtain demographic information. Medical information was obtained from the child’s medical record. The completion of the measures took approximately 45 min. Participants had the option to complete the measures in the clinic or take the packet home to complete and return in a self-addressed stamped envelope. Of the 68 participants who gave informed consent and received research questionnaires, 61 returned complete data on the instruments used in this study (an 89.7% completion rate). This number represents approximately one-third of the family caregivers served at the clinic.

Caregivers ranged in age from 24 to 60 (*M* = 43.69, SD = 8.95). Only one was male. The majority identified as Caucasian (n = 44; 72%) and the rest as Black (n = 17; 27.87%). Length of time in the caregiver role ranged from 3 to 26 years (*M* = 12.51, SD = 5.54). There were more male (n = 37) than female (n = 24) family members receiving chronic respiratory management. Twenty-five of these individuals were age 12 or less (41% of the care recipients), 24 were within the ages of 13 and 19 (39%), and 12 were within the ages of 20 and 30 (20%).

All patients at the clinic were assigned a respiratory care management code by attending medical staff reflect the specific care needs for each patient. Codes for care recipients in this study ranged from 1 (daily aerosol medications, at least one respiratory therapy device, no mechanical ventilation) to 4 (requirement of continuous mechanical ventilatory support 24 h a day utilizing a tracheostomy tube). The summary of codes for participants: 24.59% (n = 15) received a score of 1; 13.11% (n = 8) received a score of 2; 36.07% (n = 22) received a score of 3; and 26.23% (n = 16) received a score of 4.

## Measures

### Big five inventory

The Big Five Inventory (BFI; [32]) consists of 44 items rated on a 5-point Likert-type scale ranging from 1 (disagree strongly) to 5 (agree strongly). The BFI was developed to provide a short, efficient measure of the Big Five personality domains: Conscientiousness (C), Extraversion (E), Agreeableness (A), Inventiveness (the factor for Openness, O), and Worrisome (the factor representing Neuroticism, N). N was reversed-scored to facilitate interpretability (RN). The BFI is considered reliable, valid, and is widely used to measure the Big Five personality domains [33]. Test–retest reliabilities are also strong (mean of 0.85 after three months). Internal consistency coefficients for the separate traits ranged from 0.73 (for O) to 0.84 (for E).

### Coping

The Coping Health Inventory for Parents (CHIP; [34]) was administered. The CHIP contains 45 items and respondents are instructed to read the list of “coping behaviors” and record which ones they use and if yes, how helpful that coping behavior was for the person and their family (on a 3 = *extremely helpful* to 0 = *not helpful* Likert-type scale). The CHIP has three factor scores: *Cooperation and an optimistic definition of the situation* (CHIPCO; Cronbach’s* α* = 0.82; sample items: “Investing myself in my child,” “Believing my child will get better, Doing things with family relatives”), *Maintaining social support*, *self-esteem and psychological stability* (CHIPSES; *α* = 0.79; “Involvement in social activities with my friends,” “Talking to someone about how I feel,” “Building close relationships with people”), and *Understanding the medical situation through communication with other parents and consultation with the medical staff* (CHIPMCC; *α* = 0.78; e.g., “Talking with medical staff when we visit the medical center,” “Reading about how other persons in my situation handle things,” “Talking with other parents in the same type of situation and learning about their experiences”). Higher scores reflect greater usefulness on each scale.

### Resilience

The Connor-Davidson Resilience Scale (CDRS; [35]) was administered to assess self-reported resilience. Its psychometric properties are among the best in comparisons with other available resilience measures [36]. The measure contains 25 items rated on a 5-point Likert scale ranging from 0 (not true at all) to 4 (true nearly all of the time). A total score is obtained from the full 25 items (*α* = 0.91; sample items: “I am able to adapt when changes occur,” “Having to cope with stress can make me stronger”). Higher scores reflect greater self-perceived resilience.

### Physical health

The General Health (SF12GH) scale on the Short Form-12 Version 2, Health Survey [37] was used to assess caregiver physical health QoL. The full instrument has established psychometric properties and is a widely used outcome measure for quality of life in the clinical and research settings. Participants responded to the item, “In general, would you say your health is…” on a Likert-type scale ranging from “excellent” to “poor.” Reponses are transposed to a standardized score ranging from zero to 100. Higher scores on the General Health scale indicate a more optimal sense of personal health.

### Well being

The Mental Health (SF12MH) scale on the Short Form-12 Version 2, Health Survey [37] was used to assess caregiver mental health QoL. Participants responded to the items “have you felt calm and peaceful” and “have you felt downhearted and depressed” (reverse scored) on a Likert-type scale ranging from “all of the time” to “none of the time.” Standardized scores on the MH scale range from zero to 100. According to the manual, higher scores on the MH scale indicate a greater sense of well-being. These two items capture an essential component of subjective well-being by assessing the presence of positive affect and relative absence of negative affect [38].

### Statistical analysis

**Fig. 1:**
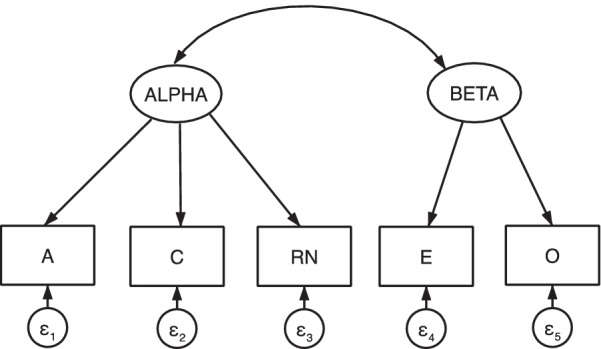
A priori CFA model of the alpha and beta metatraits. *Note*: RN, neuroticism reversed scored

DeYoung and colleagues [1, 39, 40] used confirmatory factor analysis (CFA) of the Big Five personality trait scores to operationalize the Alpha and Beta metatraits (as depicted Fig. 1). Consistent with this work, we first conducted a CFA of the Big Five trait scores to verify the Alpha and Beta metatraits for use in a subsequent path model to predict caregiver QoL.

In the CFA, items are exclusively associated with their designated factors without cross-loadings. Such a restriction might result in poor model fit because items are seldom exclusive indicators of their designated factors (e.g., [41, 42]). To overcome the potential limitation of the CFA, we used an exploratory structural equation model (ESEM) with target rotation procedure as a sensitivity analysis. ESEM with target oblique rotation is advantageous because it allows items to have cross-loadings on non-designated factors as well as the testing of a priori defined factor structure [43]. Both CFA and ESEM analyses were estimated using the maximum likelihood estimation method in Mplus 8.0 [44].

For the mediation analysis, the composite scores for the metatraits (i.e., the mean of A, C, and reverse-scored N (RN) for Alpha; the mean of E and O for Beta) were used. We first examined the correlations between all self-report variables to identify the coping variables that meet basic criteria to qualify as a mediator of the metatrait-QoL relationship [45]. Path analysis was used to test the hypothesized direct and indirect effects of the Alpha and Beta metatraits on the two indicators of caregiver QoL via coping and the self-reported resilience scale (CDRS). The path model was estimated using maximum likelihood (ML) estimation method in the Stata program [46]. Robust standard errors were used to account for potential violations of the multivariate normality assumption. Monte Carlo confidence intervals were computed for the indirect effects. We chose path analysis because multiple mediators can be tested simultaneously. However, the ML estimation method may produce biased statistical inferences when sample size is not sufficient. Hence, as a sensitivity analysis, we further conducted OLS regression analyses to estimate the indirect effects. To account for potential violations of the normality assumption, bias corrected bootstrap confidence intervals were computed for the estimated indirect effects.

## Results

Table 1 presents the means and standard deviations for all self-report variables. Correlations among all self-report variables are in Table 2. All significant correlations were in expected directions. Of the coping variables, only the CHIPSES variable was significantly correlated with both Alpha and Beta. It also significantly correlated with the two indicators of caregiver QoL. Therefore, it was the coping variable selected as a second potential mediator of the relationships between the two metatraits and the QoL outcome variables.

**Table 1:** Means, standard deviations of scores, and skewness on the self-report instruments

Variable	Possible range	Observed range	Mean	SD	Skewness	Kurtosis
*Personality traits*						
Conscientiousness	1–5	2.56–5	4.24	0.57	0.01	0.47
Agreeableness	1–5	2.33–5	4.25	0.61	0.00	0.16
Neuroticism	1–5	1.00–4.63	2.61	0.84	0.22	0.77
Extraversion	1–5	1.63–5	3.55	0.85	0.32	0.50
Openness	1–5	2.30–4.7	3.61	0.59	0.11	0.78
*CHIP scales*						
CHIPCO	0–48	18–57	44.05	9.07	0.02	0.88
CHIPSES	0–54	12–46	28.31	8.74	0.34	0.10
CHIPMCC	0–24	4–24	16.26	5.03	0.20	0.28
*Self-reported resilience*						
CDRS	0–100	28–98	74.15	16.19	0.02	0.59
*Caregiver QoL outcomes*						
SF12MH	0–100	12.5–100	74.18	17.36	0.00	0.02
SF12GH	0–100	0–100	68.52	25.94	0.01	0.66

Regular scoring was used for the neuroticism variable

**Table 2:** Correlations among self-reported variables

	1	2	3	4	5	6	7	8	9	10	11	12	13
1. A	–												
2. C	.31*	–											
3. N	− .57*	− .46*	–										
4. E	.32*	.32*	− .39*	–									
5. O	.30*	.38*	− .45*	.50*	–								
6. Alpha	.78*	.70*	− .89*	.43*	.48*	–							
7. Beta	.36*	.40*	− .48*	.91*	.81*	.52*	–						
8. CHIPSES	.24	.17	− .23	.39*	.37*	.27*	.44*	–					
9. CHIPCO	.18	.18	− .12	.14	.10	.19	.14	.63*	–				
10. CHIPMCC	.15	.12	− .01	.05	.14	.10	.10	.48*	.19	–			
11. CDRS	.18	.36*	− .34*	.27*	.32*	.37*	.34*	.17	.23	.19	–		
12. SF12MH	.46*	.25	− .61*	.18	.26*	.57*	.24	.39*	.25*	.22	.23	–	
13. SF12GH	.08	.16	− .13	.21	.25	.16	.26*	.39*	.11	.04	.18	.19	–

A, agreeableness; C, conscientiousness; N, neuroticism (regular scoring); E, extraversion; O, openness to experience (inventiveness); CHIPSES, social support, self-esteem and psychological stability; CHIPCO, cooperation and an optimistic definition of the situation; CHIPMCC, understanding the medical situation through communication; CDRS, Connor-Davidson Resilience Scale; SF12MH, SF-12 mental health; SF12GH, SF-12 general physical health

^*^p < .05

**Fig. 2:**
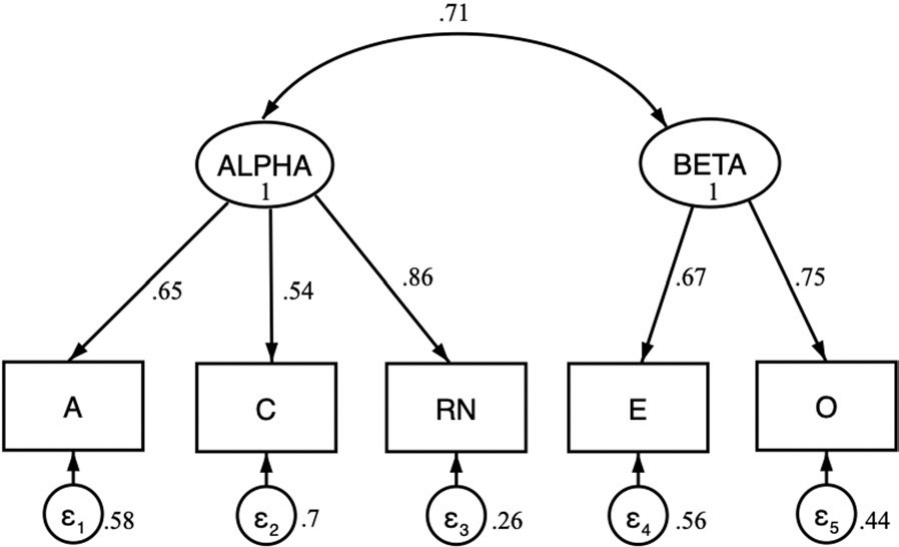
CFA model of the personality metatraits. *Note*: A, agreeableness; C, conscientiousness; RN, reversed-scored neuroticism; E, extraversion; O, openness

Table 3 contains the standardized parameter estimates and standard errors from the CFA. The calculated χ^2^ (16) = 2.01, *p* = 0.73 (*p* > 0.05), indicated good model fit. The comparative fit index (CFI) = 1.00 (CFI ≥ . 90), the Tucker-Lewis fit index (TLI) = 1.074 (TLI ≥ 0.95), and the root mean square error of approximation (RMSEA) = 0.00 (RMSEA < 0.08) also indicated a good fit between the model and the observed data. The hypothesized two higher-order personality factors were confirmed in the CFA. As depicted in Fig. 2, the factor loadings were consistent with prior research: Reversed-scored Neuroticism (conveying emotional stability), conscientiousness and agreeableness had statistically significant loadings on the Alpha factor, and extraversion and openness had statistically significant loadings on the Beta factor (*ps* < 0.05).

**Table 3:** Standardized coefficients for CFA analysis

Observed variable	Latent construct	β	*SE*	*p* <
Conscientiousness	Alpha	.545	.110	.001
Agreeableness	Alpha	.647	.094	.001
Neuroticism (reversed)	Alpha	.861	.084	.001
Extraversion	Beta	.667	.111	.001
Openness	Beta	.748	.111	.001

The results of the ESEM model were consistent with the CFA analysis (see Table 4). First, the model fit was good, χ2 (19) = 0.315, p = 0.574 (*p* > 0.05), CFI = 1.00 (CFI ≥ . 90), TLI = 1.101 (TLI ≥ 0.95), and RMSEA = 0.00 (RMSEA < 0.08). Consistent with the CFA results, Reversed-scored Neuroticism, conscientiousness and agreeableness were mainly loaded on the Alpha metatrait, and extraversion and openness were mainly loaded on the Beta metatrait.

**Table 4:** Standardized coefficients for ESEM analysis

Variable	Estimate	*SE*	*p*
*Alpha by*			
Extraversion	0.037	0.217	0.864
Agreeableness	0.578	0.528	0.274
Conscientiousness	0.314	0.262	0.232
Openness	0.051	0.310	0.869
Neuroticism (reversed)	1.026	0.498	0.039
*Beta by*			
Extraversion	0.640	0.354	0.070
Agreeableness	0.036	0.508	0.944
Conscientiousness	0.286	0.272	0.293
Openness	0.717	0.433	0.098
Neuroticism (reversed)	-0.105	0.212	0.621
Alpha with beta	0.645	0.239	0.007

**Fig. 3:**
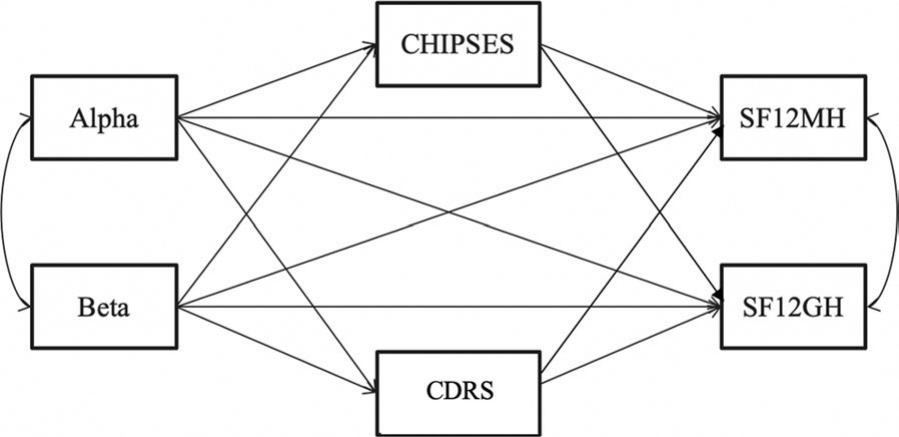
A priori path model of personality metatraits and mediating variables predicting caregiver adjustment

**Fig. 4:**
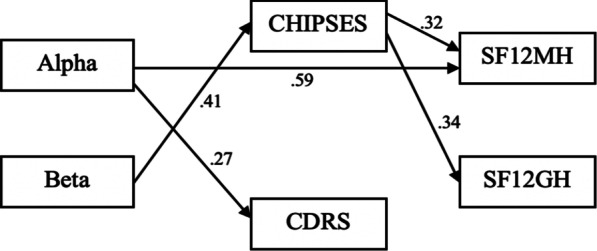
Path model of personality metatraits and mediating variables denoting significant paths

The Alpha and Beta composite scores were used as predictor variables in the path model, depicted in Fig. 3. The path model in Fig. 4 (showing only significant paths) demonstrated good model fit, χ^2^ = 0.082, *p* < 0.05. The comparative fit index (CFI) = 1.00 (CFI ≥ 0.90), the Tucker-Lewis fit index (TLI) = 1.244 (TLI ≥ 0.95), and the root mean square error of approximation (RMSEA) = 0.00 (RMSEA < 0.08) also indicated that the path model had an overall good fit. The direct and indirect path coefficients for the model are presented in Tables 5 and 6, respectively.

**Table 5:** Standardized coefficients of the path model

Dependent variable	Independent variable	Standardized estimate	Standard error	*p*
CHIPSES	Alpha	.056	.134	.676
	Beta*	.414	.122	.001
CDRS	Alpha*	.269	.099	.041
	Beta	.197	.134	.142
SF12MH	Alpha*	.587	.099	.000
	Beta	− .215	.122	.079
	CHIPSES*	.321	.106	.003
	CDRS	.033	.107	.761
SF12GH	Alpha	− .014	.141	.923
	Beta	.082	.148	.580
	CHIPSES*	.342	.124	.006
	CDRS	.097	.127	.447

^*^p < .05

**Table 6:** Indirect effect estimates from predictors to outcomes through the mediators in the path model

Effect	Unstandardized effect	Unstandardized 95% CI	Standardized effect
Alpha → CHIPSES → SF12MH	0.57	− 0.72, 1.01	0.018
Alpha → CDRS → SF12MH	0.28	− 0.52, 0.70	0.009
Beta → CHIPSES → SF12MH*	3.68	0.15, 3.54	0.132
Beta → CDRS → SF12MH	0.18	− 0.50, 0.67	0.006
Alpha → CHIPSES → SF12GH	0.92	− 1.15, 1.76	0.019
Alpha → CDRS → SF12GH	1.25	− 0.74, 1.57	0.026
Beta → CHIPSES → SF12GH*	5.87	0.06, 5.81	0.142
Beta → CDRS → SF12GH	0.79	− 0.76, 1.55	0.019

*p < .05

Considering only the statistically significant paths, the Alpha metatrait independently accounted for 23.7% of the variance in SF12MH, and 13.8% of the variance in the Connor-Davidson Resilience variable (the CDRS; *R*^2^ = 0.138). The Beta metatrait accounted for 19.6% of the variance in the parental coping style to maintain social support, self-esteem, and psychological stability (*R*^2^ = 0.196). Beta and CHIPSES combined accounted for 31.4% of the variance in mental health (*R*^2^ = 0.314) and 38.3% of the variance in physical health (*R*^2^ = 0.383).

The regression analyses resulted in the same parameter estimates and consistent statistical inferences as the path analysis, indicating that the results are not sensitive to the analytical approaches. The regression analyses results are reported in the online supplemental materials.

In sum, these results indicate that Alpha had a specific effect on positive mental health among caregivers, presumably due, in part, to the emotional stability and self-regulation that characterize this metatrait. This association was independent of the mediating variables. It was not, however, predictive of caregiver physical health QoL. In contrast, Beta had no direct effect on either outcome variable, but indirect effect estimates reveal that it exerted beneficial effects through the coping variable to caregiver physical and mental health QoL. Theoretically, the adaptive flexibility, resourcefulness and social engagement associated with the Beta metatrait may have influenced caregiver QoL through its positive association with coping efforts to maintain social support, self-esteem and family stability.

## Discussion

Contrary to our original expectations, our analytic model presents a more straightforward, albeit theoretically consistent, picture of caregiver personality and resilience. Higher Alpha scores among caregivers were directly associated with their emotional well-being. But the Beta metatrait was predictive of both physical and mental health through its beneficial association with caregiver coping. Few studies examine coping as a mediating variable in this literature [13], and our findings underscore the value of analytic models that conceptualize coping as a mediating factor in predicting adjustment.

The significant association of Alpha with caregiver mental health may be attributable, in part, to the known relationship of neuroticism to distress, negative cognitions, and stress, generally [47]. In DeYoung’s [48] theoretical model of the metatraits, Alpha is recast as “Stability,” which appropriately conveys its properties that regulate motivations, behavior, perceptions and cognitions to engage in meaningful goal pursuits. These self-regulatory properties serve to manage negative emotions and cognitions, but they also apply to characteristics associated with Agreeableness and Conscientiousness to promote motivational and social stability [48]. Consistent with prior research, N had the largest contribution to Alpha, but the contributions of A and C are critical to understanding the properties of this metatrait. High A and C likely endow caregivers with capacities essential to engaging in goal-directed behavior, attending to tasks, and maintaining interpersonal and social relationships, without being disrupted by prolonged negative moods or cognitions [39, 48].

Beta exerted its effects on both elements of QoL through its beneficial association with caregiver coping efforts to “…maintain a sense of their own well-being through social relationships, involvement in activities that have the potential of enhancing one’s self-esteem, and doing things to manage psychological tensions and strains” (pp. 363; [49]).

In DeYoung’s model [48] of the two metatraits, Beta is understandably labeled as ‘Plasticity,’ denoting its linkage to the Lewinian concept and terminology. Its adaptable flexibility enables an individual to identify new goals and strategies in the face of new information, stress, and interpersonal, social and other environmental demands [48]. Feldman [50] argues that Plasticity is essential for the coordinated social behavior necessary for personal resilience. In contrast, individuals lower on this metatrait are likely seen as rigid, struggling to manage and accommodate challenges they encounter [48]. Our findings suggest that caregivers with higher Beta scores were resourceful, coping in ways that facilitated their physical and mental health-related QoL.

Other evidence indicates that the coping behavior included in our model is associated with caregiver adjustment. Boettcher et al. [18] found this coping style significantly predicted the mental health and quality of life of mothers of children receiving long-term mechanical ventilation. A study of parents of children with neurological disorders found it was the only coping style that mediated the relationships of four distinct illness perceptions—personal control, perceived consequences of the child’s illness, treatment control and perceived longevity of the illness—to caregiver depression [14].

We assumed both metatraits would be positively associated with self-reported resilience. Only Alpha had a significant relationship with the CDRS total score. Further, self-reported resilience did not contribute to the prediction of either QoL variable. As in the present study, Farkas and Orosz [51] found the Connor-Davidson instrument was significantly associated with the Alpha metatrait, but not with Beta (they used the terms Stability and Plasticity, respectively). The association of the CDRS to quality of life may be influenced by an array of factors including the nature of the clinical sample, methodological features (the design, the outcome measures studied, etc.), and sample size.

### Limitations

Tests of mediation, by definition, assume some temporal sequencing of effects between the predictor, mediating and outcome variables that occur over time [52, 53]. Cross-sectional designs, such as ours, do not adequately test mediation effects. Although we relied on a coherent and reasonable theoretical model to guide our understanding of the relationships between the variables measured, cross-sectional designs can still produce biased results that can “over- or underestimate effects” ([52], p. 40). Prior longitudinal studies of a resilient personality prototype (configured from the Big Five traits) have found inconsistent evidence of the mediating effects of self-reported resilience in predicting post-traumatic stress symptoms, depression and quality of life among warzone veterans [54, 55], and in the prediction of distress among health care workers [56]. In contrast, these same studies consistently found the relationship of a resilient personality prototype to these outcome variables was mediated by a lower use of avoidant coping.

Although our sample size is consistent with previous clinical studies of family caregivers who provide chronic respiratory management for dependent children and adolescents [10], the relatively low number of participants in our study warrants caution in interpreting our results. Our findings are consistent with past studies of the two metatraits and their relationship to well-being [31]. Path modeling was the most appropriate method to test the relation of the two metatraits to the two outcome variables in the context of two mediating variables [57], and our post hoc regression analyses were consistent with our path model results. We believe the sample size was sufficient to have stable estimates in the path model [58], and we hope our results demonstrate the importance of theory-driven investigations with understudied clinical samples living with low incidence and medically complex conditions [59].

The preponderance of women in the sample reflects the reality of caregiving in this clinical scenario, further exposing the lack of information we have about fathers who assume a primary caregiving role. There are other clinical realities about the sample to consider. Individuals served at the clinic have demonstrated an ongoing commitment to the child, and to their caregiving activities and responsibilities. It is unlikely that parents who were unable or unwilling to commit to the caregiver role were among the rather select and unique group of individuals at the clinic who learned about our study. It is also possible that participants felt compelled to respond in a socially desirable manner, to appear competent and capable of managing their role. Additionally, future studies may prefer to use other measures of quality of life with more items than the scales we selected from the SF-12 (one item for SF12GH, two for SF12MH).

In sum, the limitations of the present study restrict our interpretations of the results. Perhaps our findings may be regarded as a possible indication that the DeYoung conceptualization of the two personality metatraits may be useful in advancing our understanding of resilience in chronic and potentially stressful episodes.

## Conclusions

In our study, we defined resilience in positive terms, using non-pathological personality traits to predict quality of life (rather than infer resilience from an absence or lower than expected level of distress). Our findings suggest that the two metatraits, Alpha and Beta, may prove important to our theoretical understanding of resilience, generally. Despite their pervasive presence in the personality literature, the Big Five traits are not typically assessed in psychological evaluations conducted in these clinical settings [60]. Interestingly, they are listed in the World Health Organization’s International Classification of Functioning, Disability and Health (WHO-ICF) as personal factors clinicians could use to determine patient and family members’ unique strengths and vulnerabilities (under “b126 Temperament and Personality Factors;” https://apps.who.int/classifications/icfbrowser/). The inclusion of these five personality traits in the WHO-ICF reflects a general appreciation of their value in clinical practice. Perhaps our findings will prompt others to consider the two metatraits and their properties as potential indicators of personal resilience. An overarching model of resilient personality characteristics may help us anticipate important, a priori differences among individuals in caregiving roles in ways a unidimensional model of resilience cannot, particularly in scenarios in which commonplace personality traits may predominate and psychopathological characteristics are less likely to be a concern.

## Supplementary Information

**Additional file 1.** This file contains tables containing the results of the regression analysis of the a priori model of the metatraits, the mediators, and the two quality of life outcome variables.

## Data Availability

Deidentified data generated during and analysed during the current study are not publicly available, but are available from the corresponding author on reasonable request to the second author and with the permission of the Texas A&M IRB. The original application for IRB approval (IRB #110880) did not request approval to make the data publicly available. Participants were not informed of this possibility, and they did not consent to it.

## Abbreviations

A: Agreeableness
BFI: Big five inventory
C: Conscientiousness
CDRS: Connor-Davidson Resilience Scale
CFI: Comparative Fit Index
CHIP: Coping health inventory for parents
CHIPCO: Cooperation and Optimistic Definition of the Situation Scale
CHIPMCC: Understanding the Medical Situation Through Communication with Parents and Medical Staff Scale
CHIPSES: Maintaining Social Support, Self-Esteem and Psychological Stability Scale
E: Extraversion
ESEM: Exploratory structural equation model
N: Neuroticism
O: Openness to experience
QoL: Quality of life
RMSEA: Root mean squared error of approximation
RN: Reversed Neuroticism Score
SF12GH: General Health Scale on the Short Form-12 Version 2, Health Survey
SF12MH: Mental Health Scale on the Short Form-12 Version 2, Health Survey
TLI: Tucker-Lewis Index

## References

[1] DeYoung CG (2010). Toward a theory of the Big Five. Psychol Inq.

[2] Heaton J, Noyes J, Sloper P, Shah R (2005). Families’ experiences of caring for technology-dependent children: a temporal perspective. Health Soc Care Community.

[3] Primhak RA, Hicks B, Shaw NJ, Donaldson GC, Balfour-Lynn IM (2011). Use of home oxygen for children in England and Wales. Arch Dis Child.

[4] Westbom L, Bergstrand L, Wagner P, Nordmark E (2011). Survival at 19 years of age in a total population of children and young people with cerebral palsy. Dev Med Child Neurol.

[5] Carnevale FA, Alexander E, Davis M, Rennick J, Troini R (2006). Daily living with distress and enrichment: the moral experience of families with ventilator-assisted children at home. Pediatrics.

[6] Sarvey SI (2008). Living with a machine: the experience of the child who is ventilator dependent. Issues Ment Health Nurs.

[7] Alro AB, Klitnaes C, Rossau CD, Dreyer P (2021). Living as a family with a child on home mechanical ventilation and personal care assistants—a burdensome impact of family life. Nurs Open.

[8] Keilty K, Daniels C (2018). CTS pediatric home ventilation guidelines panel. Section 13: the published experience and outcomes of family caregivers when a child is on home mechanical ventilation. Can J Respir Crit Care Sleep Med..

[9] Toledano-Toledano F, Dominguez-Guedea MT (2019). Psychosocial factors related with caregiver burden among families with children with chronic conditions. BioPsychoSoc Med.

[10] Lee J, Lynn F (2017). Mental health and well-being of parents caring for a ventilator-dependent child. Nurs Child Young People.

[11] Brehaut JC, Garner RE, Miller AR, Lach LM, Klassen AF, Rosenbaum PL, Kohen DE (2011). Changes over time in the health of caregivers of children with health problems: growth-curve findings from a 10-year Canadian population-based study. Am J Public Health.

[12] Chan YH, Lim CZR, Bautista D, Malhotra R, Ostbye T (2019). The health and well-being of caregivers of technologically dependent children. Glob Pediatr Health.

[13] Fairfax A, Brehaut J, Colman I, Sikora L, Kazakova A, Chakraborty P, Potter BK (2019). A systematic review of the association between coping strategies and quality of life among caregivers of children with chronic illness and disability. BMC Pediatr.

[14] Kelada L, Wakefield EC, Muppavaram N, Lingappa L, Chittem M (2021). Psychological outcomes, coping and illness perceptions among parents of children with neurological disorders. Psychol Health.

[15] Windle G, Bennett KM. Caring relationships: how to promote resilience in challenging times. In: Unger M, editor. The social ecology of resilience: a handbook of theory and practice. Springer. 2012. p. 219–231.

[16] Davydov DM, Stewart R, Ritchie K, Chaudieu I (2010). Resilience and mental health. Clin Psychol Rev.

[17] Andrews EE, Dunn RA. Families and disability. In: Brenner LA, Reid-Arndt SA, Elliott T, Frank RG, Caplan B, editors. Handbook of rehabilitation psychology, 3rd edition. American Psychological Association. 2019. p. 189–202.

[18] Boettcher J, Denecke J, Barkmann C, Wiegand-Grefe S (2020). Quality of life and mental health in mothers and fathers caring for children and adolescents with rare diseases requiring long-term mechanical ventilation. Int J Environ Res Public Health.

[19] Glidden LM, Natcher AL (2009). Coping strategy use, personality, and adjustment of parents rearing children with developmental disabilities. J Intellect Disabil Res.

[20] Glidden LM, Billings FJ, Jobe BM (2006). Personality, coping styles and well-being of parents rearing children with developmental disabilities. J Intellect Disabil Res.

[21] Hajek A, König H-H (2018). The relation between personality, informal caregiving, life satisfaction and health-related quality of life: evidence of a longitudinal study. Qual Life Res.

[22] Digman JM (1997). Higher-order factors of the Big Five. J Personal Soc Psychol.

[23] Löckenhoff CE, Duberstein PR, Friedman B, Costa PT (2011). Five-factor personality traits and subjective health among caregivers: the role of caregiver strain and self-efficacy. Psychol Aging.

[24] Block JH, Block J. The role of ego control and ego resiliency in the organization of behavior. In: Collins WA. editor. The Minnesota symposium on child psychology, vol. 13. Development of cognition, affect, and social relations. Hillsdale NJ. 1980. p. 39–101.

[25] Lewin K. Principles of topological psychology. 1936. McGraw-Hill.

[26] Caspi A (2000). The child is father of the man: personality continuities from childhood to adulthood. J Pers Soc Psychol.

[27] Dennissen JJA, Asendorpf JB, Van Aken MAG (2007). Childhood personality predicts long-term trajectories of shyness and aggressiveness in the context of demographic transitions in emerging adulthood: long-term trajectories. J Pers.

[28] Ong AD, Bergeman CS, Boker SM (2009). Resilience comes of age: defining features in later adulthood. J Pers.

[29] Strus W, Cieciuch J (2017). Towards a synthesis of personality, temperament, motivation, emotion and mental health models within the circumplex of personality metatraits. J Res Pers.

[30] Strus W, Cieciuch J, Rowinski T (2014). A synthesizing model of personality based on the big five. Rev Gen Psychol.

[31] Mann FD, DeYoung CG, Tiberious V, Krueger R (2021). Stability and well-being: associations among the Big Five domains, metatraits, and three kinds of well-being in a large sample. J Pers.

[32] John OP, Srivastava S, Pervin LA, John OP (1999). The Big Five trait taxonomy: history, measurement, and theoretical perspectives. Handbook of personality: theory and research.

[33] John OP, Naumann LP, Soto CJ, John OP, Robins RW, Pervin LA (2008). Paradigm shift to the integrative big five trait taxonomy: history, measurement, and conceptual issues. Handbook of personality: theory and research.

[34] McCubbin HI, McCubbin MA, Patterson JM, Cauble AE, Wilson LR, Warwick WCHIP (1983). Coping health inventory for parents: an assessment of parental coping patterns in the care of the chronically ill child. J Marriage Fam.

[35] Connor KM, Davidson JR (2003). Development of a new resilience scale: The Connor-Davidson Resilience Scale (CD-RISC). Depress Anx.

[36] Windle G, Bennett KM, Noyes J (2011). A methodological review of resilience measurement scales. Health Qual Life Outcomes.

[37] Ware JE, Kosinski M, Turner-Bowker D, Gandek B. Version 2 of SF-12 Health Survey. Quality Metric Inc. 2002.

[38] Diener E, Kesebir P, Tov W, Leary MR, Hoyle RH (2009). Happiness. Handbook of individual differences in social behavior.

[39] DeYoung CG (2006). Higher-order factors of the Big Five in a multi-informant sample. J Pers Soc Psych.

[40] DeYoung CG, Peterson JB, Higgins DM (2002). Higher-order factors of the Big Five predict conformity: Are there neuroses of health?. Pers Individ Differ.

[41] Gomez R, Stavropoulos V, Griffiths MD (2020). Confirmatory factor analysis and exploratory structural equation modelling of the factor structure of the Depression Anxiety and Stress Scales-21. PLoS ONE.

[42] Morin AJ, Arens AK, Tran A, Caci H (2016). Exploring sources of construct-relevant multidimensionality in psychiatric measurement: a tutorial and illustration using the Composite Scale of Morningness. Int J Methods Psychiatr Res.

[43] Asparouhov T, Muthén B (2009). Exploratory structural equation modeling. Struct Equ Modeling.

[44] Muthén LK, Muthén BO. Mplus user's guide, 8th Ed*.* Los Angeles, CA: Muthén & Muthén. 1998–2017.

[45] Baron RM, Kenny DA (1986). The moderator–mediator variable distinction in social psychological research: conceptual, strategic, and statistical considerations. J Pers Soc Psychol.

[46] StataCorp. Stata Statistical Software: Release 16. StataCorp LP. 2019.

[47] Suls J, Martin R (2005). The daily life of the garden-variety neurotic: reactivity, stressor exposure, mood spillover, and maladaptive coping. J Pers.

[48] DeYoung CG (2015). Cybernetic big five. J Pers Res.

[49] McCubbin MA, McCubbin HI, McCubbin HI, Thompson A, McCubbin MA (1996). Resiliency in families: a conceptual model of family adjustment and adaptation in response to stress and crises. Family assessment: resiliency coping and adaptation inventories for research and practice.

[50] Feldman R (2021). Social behavior as a transdiagnostic marker of resilience. Annu Rev Clin Psychol.

[51] Farkas D, Orosz G (2015). Ego-resiliency reloaded: a three-component model of general resiliency. PLoS ONE.

[52] Maxwell SE, Cole DA (2007). Bias in cross-sectional analyses of longitudinal mediation. Psychol Methods.

[53] O’Laughlin KD, Martin MJ, Ferrer E (2018). Cross-sectional analysis of longitudinal mediation processes. Multivariate Behav Res.

[54] Elliott TR, Hsiao Y-Y, Kimbrel N, Meyer E, DeBeer B, Gulliver S, Kwok OM, Morissette S (2015). Resilience, traumatic brain injury, depression, and posttraumatic stress among Iraq/Afghanistan war veterans. Rehabil Psychol.

[55] Elliott TR, Hsiao YY, Kimbrel NA, DeBeer B, Gulliver SB, Kwok OM, Morissette S, Meyer EC (2019). Resilience facilitates adjustment through greater psychological flexibility among Iraq/Afghanistan war veterans with and without mild traumatic brain injury. Rehabil Psychol.

[56] Elliott TR, Perrin PB, Bell A-S, Powers M, Warren AM (2021). Resilience, coping, and distress among healthcare service personnel during the COVID-19 pandemic. BMC Psychiatry.

[57] Mueller R. Basic principles of structural equation modeling. New York. 1996.

[58] MacCallum RC, Austin JT (2000). Applications of structural equation modeling in psychological research. Ann Rev Psychol.

[59] Bradshaw S, Bem D, Shaw K, Taylor B, Chiswell C, Salama M, Bassett E, Kaur G, Cummins C (2019). Improving health, wellbeing and parenting skills in parents of children with special health care needs and medical complexity—a scoping review. BMC Pediatr.

[60] Elliott TR, Wade L, Dong S, Budge K, Wehmeyer M, Dunn D (2021). Personality and disability. The positive psychology of personal factors: implications for understanding disability.

