# Supplementary material for: Personality metatraits predict resilience among family caregivers responsible for a dependent youth’s chronic respiratory management
Source: BMC Psychol. 2022 Apr 1;10:85. doi: 10.1186/s40359-022-00791-y (PMC8973997; doi:10.1186/s40359-022-00791-y)
Supplement: Supplementary file 1 — Additional file 1. This file contains tables containing the results of the regression analysis of the a priori model of the metatraits, the mediators, and the two quality of life outcome variables. [file 40359_2022_791_MOESM1_ESM.docx]

Table 1

*Standardized Coefficients of the Regression Analysis*

­Dependent Independent B S.E. β *p*

Variable Variable

CHIPSES Alpha .902 2.22 .056 .686

Beta* 5.78 .414 .122 .004

CDRS Alpha* 8.04 4.19 .269 .049

Beta 5.09 3.58 .197 .160

SF12MH Alpha* 18.79 3.42 .587 .001

Beta -5.97 3.75 -.215 .118

CHIPSES* .638 .237 .321 .009

CDRS .035 .115 .033 .763

SF12GH Alpha -.650 6.70 -.014 .923

Beta 3.40 6.74 .082 .616

CHIPSES* 1.02 .410 .342 .016

CDRS .155 .200 .097 .442

* *p* < .05

Table 2

*Indirect Effect Estimates from Predictors to Outcomes through the Mediators based on Regression Analyses*

Effect Unstandardized Unstandardized Standardized

Effect 95% Bootstrap CI Effect

Alpha 🡪 CHIPSES 🡪 SF12MH 0.58 -2.29, 3.52 0.048

Alpha 🡪 CDRS 🡪 SF12MH 0.28 -1.73, 2.39 0.55

Beta 🡪 CHIPSES 🡪 SF12MH* 3.69 0.59, 7.43 0.172

Beta 🡪 CDRS 🡪 SF12MH 0.18 -1.25, 1.72 0.006

Alpha 🡪 CHIPSES 🡪 SF12GH 0.92 -3.72, 5.87 0.054

Alpha 🡪 CDRS 🡪 SF12GH 1.25 -2.06, 5.45 0.019

Beta 🡪 CHIPSES 🡪 SF12GH* 5.87 0.77, 12.15 0.194

Beta 🡪 CDRS 🡪 SF12GH 0.79 -1.59, 3.92 0.022

* *p* < .05
